# Inflammatory Paradental Cyst: A Case Report

**DOI:** 10.7759/cureus.71405

**Published:** 2024-10-13

**Authors:** N. Fazulunnisa Begum, Karthikeyan Ramalingam, Pratibha Ramani, Senthil Murugan P

**Affiliations:** 1 Oral Pathology and Microbiology, Saveetha Dental College and Hospitals, Saveetha Institute of Medical and Technical Sciences, Saveetha University, Chennai, IND; 2 Oral and Maxillofacial Surgery, Saveetha Dental College and Hospitals, Saveetha Institute of Medical and Technical Sciences, Saveetha University, Chennai, IND

**Keywords:** cyst, inflammation, inflammatory odontogenic cyst, lateral, mandible, molars, odontogenic cyst, paradental, periodontal, vital tooth

## Abstract

Inflammatory paradental cysts (IPC) are frequently under-reported due to insufficient clinical details. Our case report describes a 45-year-old male with a complaint of discomfort in the right lower posterior region. Intra-oral examination revealed a partially erupted, mesioangularly impacted 48 without any dental caries. The radiograph revealed a well-circumscribed radiolucency around the distal root of the impacted mandibular third molar. Histopathology revealed odontogenic epithelium with an inflamed connective tissue wall. It was diagnosed as an IPC correlating the clinical and radiological findings. This case report describes the importance of clinical correlation to diagnose IPC.

## Introduction

An inflammatory paradental cyst (IPC) is an infrequent odontogenic cyst, with a prevalence ranging from 1% to 5%. It is typically associated with inflammation around a partially erupted or impacted tooth, most commonly the mandibular third molar [1, 2]. IPCs are more prevalent in young adults, particularly between the ages of 20 to 30, with a slight male predominance noted in the literature. Although the exact incidence is difficult to determine, IPCs are considered less common than other odontogenic cysts such as dentigerous or radicular cysts [3, 4].

The mandibular third molar region remains the most frequent site of occurrence, often linked to chronic pericoronitis. Radiographically, the lesions typically appear as well-defined radiolucencies superimposed on the buccal root surface but with an intact lamina dura surrounding the roots, and with no evidence of widening of the periodontal ligament space [1, 3-5]. There is no evidence in the literature to suggest malignant transformation of IPCs. It is distinct from other cystic lesions like ameloblastoma and odontogenic keratocysts (OKC) with higher recurrence rates and the potential for aggressive behavior is important [6].

The criteria proposed by Philipsen et al. [7] are as follows: IPC frequently involves erupting mandibular 3rd molar on the buccal and/or distal to the root (not the crown). Irrespective of the location of the IPC, the associated tooth must be partly or fully erupted. Completely embedded or impacted teeth with a pericoronal cystic lesion are disregarded. Although a vital pulp of the involved tooth (evaluated by electrometrical testing) by most authors is regarded as an important diagnostic criterion. If the vitality test suggests a non-vital pulp, the diagnosis of a lateral radicular cyst should be considered rather than an IPC. The origin of the epithelial lining of the IPC is still speculative. Association with mandibular molars seems to be a characteristic clinical feature of the IPC. With regard to IPC/3rd mandibular molar, removal of the molar with enucleation of the cyst is the treatment of choice.

We present a case of an inflammatory paradental cyst involving the mandibular third molar with its clinical, radiological, and histopathological findings.

## Case presentation

**Figure 1:**
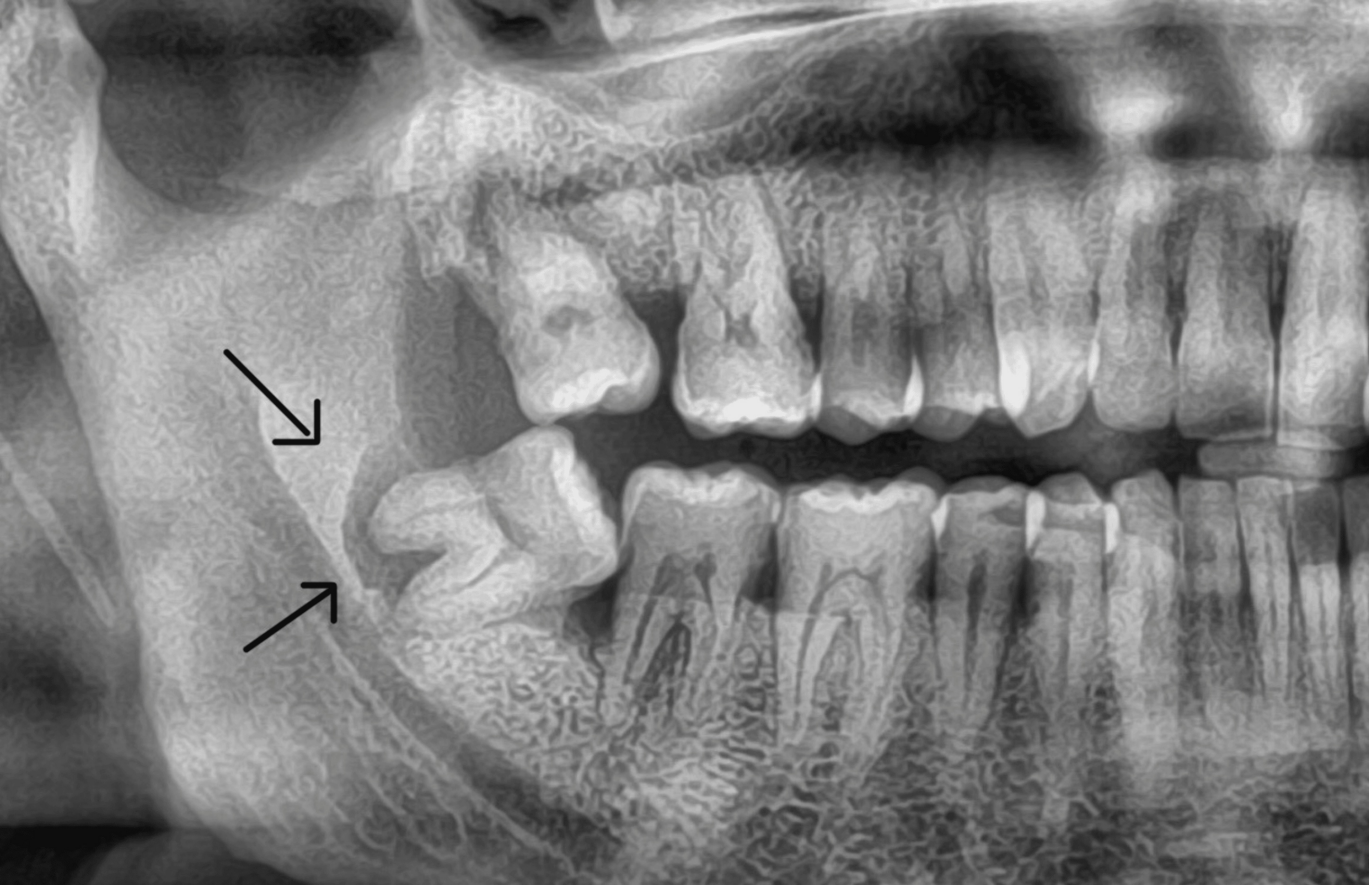
Radiograph showing well-defined radiolucency involving the distal root of mesioangular impacted 48. There is bone loss related to the distal aspect of 47 and the mesial aspect of 48.

A 45-year-old male patient reported to the Saveetha Dental College and Hospitals with a complaint of pain in the lower right back tooth region for the past few weeks. The patient was healthy with no systemic complaints. The past medical history, surgical history, and dental history were non-contributory. There were no obvious extraoral findings. On intraoral examination, there was a partially erupted mandibular third molar (48). The tooth did not have any visible signs of dental caries and its vitality was confirmed with electrical pulp testing. The pericoronal soft tissues were inflamed, bled on probing, and tender on palpation. Radiological examination revealed a well-circumscribed radiolucency surrounding the distal root of mesioangular impacted 48 extending to the furcation area but with intact periodontal ligament space noted in the mesial root. Bone loss related to the distal aspect of 47 and the mesial aspect of 48 was noted (Figure 1).

Based on the clinical and radiological examination, a provisional diagnosis was given as an odontogenic cyst - dentigerous cyst. An excisional biopsy was performed under local anesthesia. The excised soft tissue along the impacted tooth (48) was submitted to the oral pathology department for reporting.

**Figure 2:**
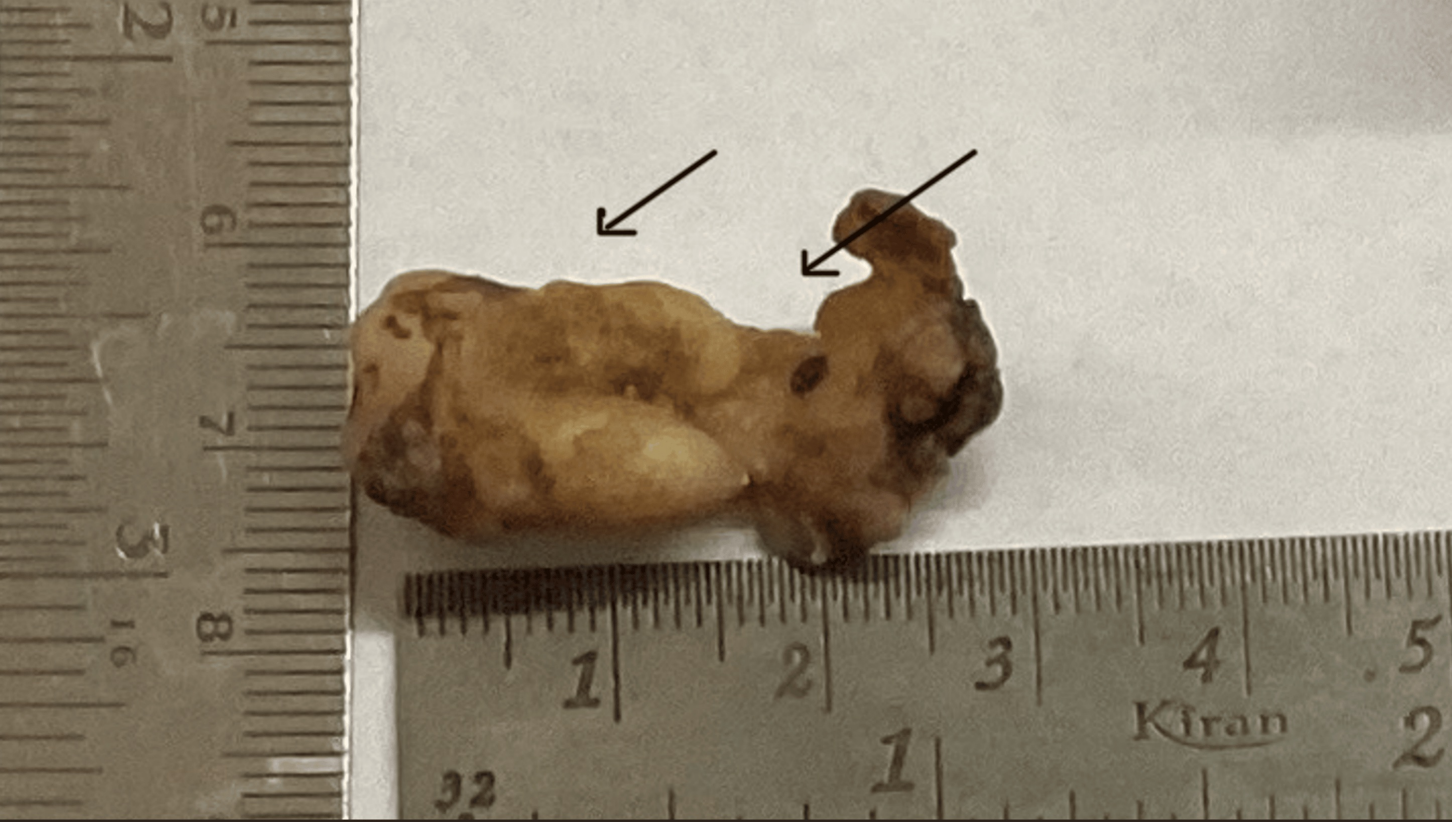
Photograph showing the gross image of the specimen with soft tissue attached to the distal root of 48.

A single formalin-fixed soft tissue specimen along with the mandibular molar was received. It measured 3.9 x 1.3 x 0.1 cm in size. Soft tissue was attached to the entire distal root of 48 (Figure 2).

**Figure 3:**
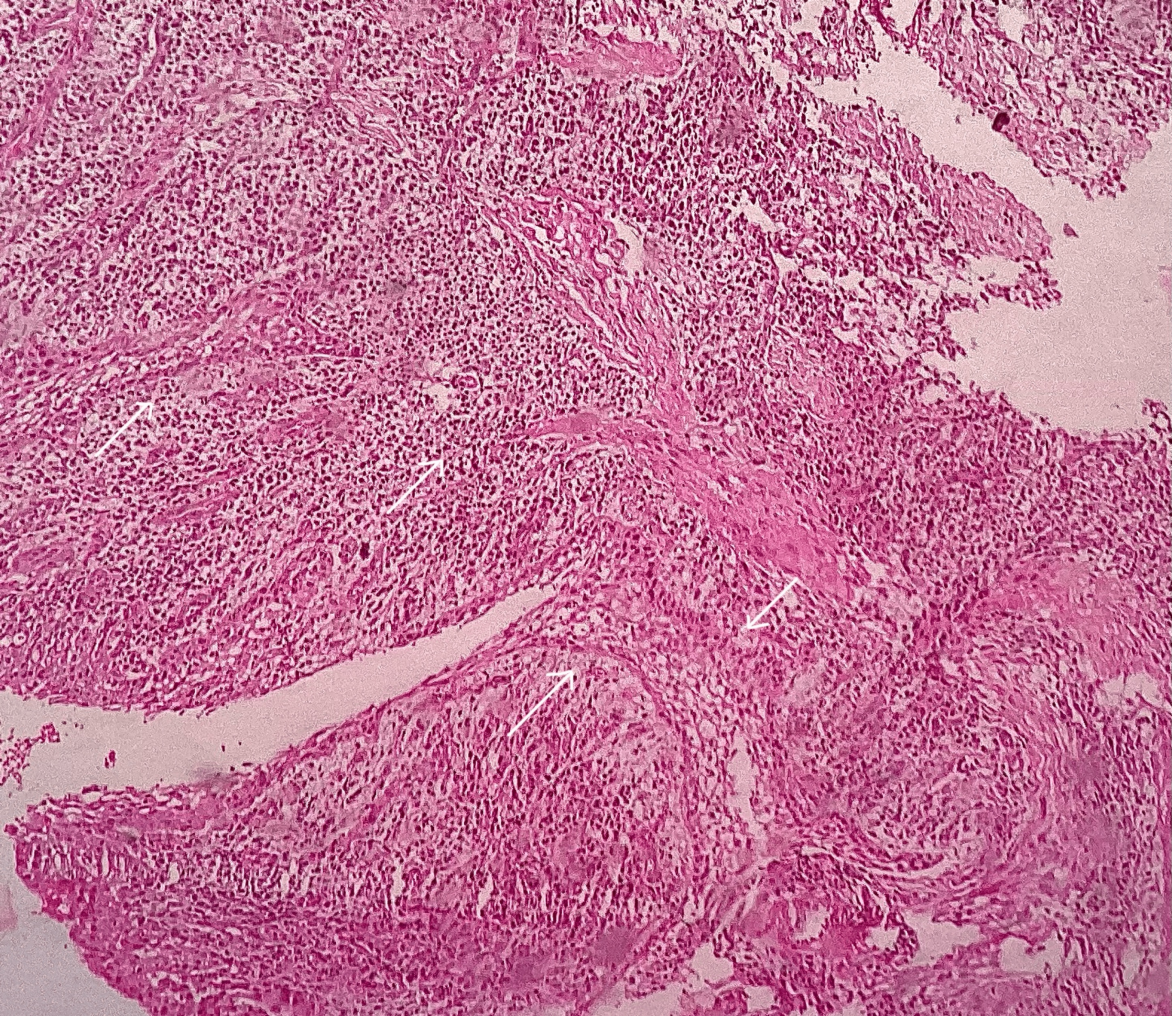
Photomicrograph Photomicrograph showing odontogenic epithelial lining and connective tissue wall with intense inflammation (H&E, 10x). White arrows represent the proliferation of non-keratinized stratified squamous epithelium - odontogenic epithelial lining.

Histopathological sections showed odontogenic epithelial lining and connective tissue wall (Figure 3). The odontogenic epithelial lining was a non-keratinized stratified squamous epithelium of variable thickness. In a few areas, there was hyperplasia and spongiosis noted in the epithelium. The connective tissue wall showed intense inflammatory cell infiltration predominantly lymphocytes and extravasated RBCs along with foci of moderate vascularity. There was no evidence of keratin, theques, palisading basal cells, or stellate reticulum-like cells in the epithelial lining.

Correlating the clinical and radiological findings, histopathology was suggestive of inflammatory paradental cyst (IPC). The post-operative healing was satisfactory and the patient remained asymptomatic on the 18-month follow-up.

## Discussion

An inflammatory paradental cyst (IPC) is usually associated with a vital tooth. In contrast to other inflammatory cysts, such as radicular cysts, which are associated with non-vital teeth resulting from pulp necrosis, IPCs develop due to inflammation surrounding a partially erupted or impacted tooth most often the mandibular third molar where the tooth itself remains vital. The inflammation primarily affects the periodontal tissues surrounding the tooth rather than the pulp [3-6].

IPC is also called an inflammatory collateral cyst or mandibular infected buccal cyst. In 1930, Hofrath introduced the term "marginal wisdom tooth cyst" [7, 8]. In 1976, Craig reported odontogenic cysts involving the lateral aspect of roots of partially erupted mandibular third molars that also showed pericoronitis [9]. In 1992, the World Health Organization (WHO) classified inflammatory odontogenic cysts into two categories: radicular cysts and paradental cysts [10]. Vedtofte and Holmstrup in 1989 and Morimoto et al. in 2004 described both paradental and juvenile paradental cysts [5, 11]. Philipsen et al. in 2004 confirmed the criteria for diagnosis of IPC [7].

The pathogenesis of paradental cysts is thought to arise from the downward extension of the reduced enamel epithelium around the tooth crown or proliferation of the cell rests of Malassez. It can also form due to periodontal destruction and unilateral expansion of the dental follicle. The IPC lining could be attached to cementoenamel junction (CEJ) mimicking dentigerous cysts and can also continue with oral epithelium. Additionally, paradental cysts are sometimes called as eruption pocket cysts, where occlusion of the pocket opening by debris accumulation leads to inflammation. Craig suggested that cyst growth may occur through osmotic pressure, akin to the mechanisms described for radicular cysts [9].

According to the World Health Organization (WHO), the term “paradental cyst” is a cyst that forms near the cervical margin on the lateral side of the root due to an inflammatory process within the periodontal pocket [4]. The distinct clinical findings of impacted tooth, pericoronitis, and association with a vital tooth were key diagnostic features of IPC in our patient. The radiological findings helped to distinguish it from the dentigerous cyst (radiolucency around the crown) and the radicular cyst (radiolucency around the root apex with loss of lamina dura). Histopathological analysis confirmed the diagnosis of IPC. More aggressive lesions like OKC or ameloblastoma can present as a single radiolucency associated with a tooth but can be ruled out with classic histopathological features [6, 7].

Misdiagnosis of ameloblastoma or odontogenic keratocyst can lead to radical surgery [12-15]. Lateral periodontal cysts could mimic IPC and can be distinguished by histopathology [16]. The recommended treatment of IPC typically involves surgical enucleation of the cyst, often accompanied by the removal of the associated tooth if necessary. This case emphasizes prompt diagnosis and management to avoid secondary infections, bone loss, and damage to adjacent teeth or vital structures.

## Conclusions

Inflammatory paradental cysts are rare odontogenic cysts involving partially erupted teeth. Our case report details a 45-year-old male who presented with discomfort in the right lower posterior area. An intraoral examination identified a mesioangularly impacted 48 without any signs of dental caries. Radiographic analysis showed a well-defined radiolucency surrounding the roots of the partially erupted mandibular third molar. Histopathological examination revealed odontogenic epithelium with an inflamed connective tissue wall. The findings led to a diagnosis of an inflammatory paradental cyst. It was treated with the removal of the involved third molar with the cystic lesion.
